# Distinctive Acellular Lipid Emboli in Hemoglobin SC Disease following Bone Marrow Infarction with Parvovirus Infection

**DOI:** 10.1155/2015/328065

**Published:** 2015-08-27

**Authors:** Danielle M. Graff, Erin Owen, Robert Bendon, Salvatore Bertolone, Ashok Raj

**Affiliations:** ^1^University of Louisville, 571 S. Floyd Street, Suite 300, Louisville, KY 40202, USA; ^2^Pathology, Norton Healthcare, Louisville, KY, USA

## Abstract

An adolescent with mild hemoglobin SC disease presented with pelvic pain with subsequent respiratory and neurologic deterioration, which led to ultimately death. The autopsy demonstrated acellular fat emboli particularly in the lung and brain. There was marrow necrosis in the lumbar spine with aggregated sickle cells and positive parvovirus immunostaining. The brain lesion both grossly and microscopically presented a distinct pathology of acellular fat emboli that led to the correct diagnosis of this increasingly recognized association of sickle hemoglobinopathies with fat embolism syndrome (FES). A clinical diagnosis of FES is difficult to confirm in many patients with sickle hemoglobinopathy presenting with pain crisis because of concurrent illness. However, this case report highlights the need for a thorough knowledge of the signs and symptoms of the syndrome and a high index of suspicion for the diagnosis to be made premortem.

## 1. Introduction

Fat embolism syndrome (FES) is a rare but known serious complication of considerable bone marrow necrosis, especially when confounded with sickle cell disease (SCD) [1–3]. With its rapidly progressive multisystem involvement and range of symptoms including respiratory distress, neurologic changes, and fever, the diagnosis may be vague often leading to a late discovery. Though FES has been associated with bone marrow necrosis in patients with hemoglobinopathies, it is often not considered in the primary differential diagnosis of acute complications in children with SCD.

The etiology of bone marrow necrosis, which may lead to FES, has continuously been linked to parvovirus B19 (B19V) [3–7]. However, B19V can also be linked to other serious illnesses such as transient aplastic crises in patients with hemolytic anemia and hydrops fetalis in midtrimester pregnancies. As a result of the cytotoxic effects, tissue hypoxia and microvascular endothelial injury resulting in end organ failure are noted.

We report a patient with HbSC disease who developed fatal fat emboli in association with B19V infection following an erythrocytapheresis.

## 2. Case Report

The patient was an 18-year-old African American female with modestly controlled hemoglobin SC disease. She presented with severe lower back and bilateral leg pain. Her medical history was unremarkable except for a limited number of admissions for pain crisis, a positive sexual history, and a recent tattoo obtained two weeks prior to admission. Four days prior to presentation, she had undergone an elective erythrocytapheresis procedure in preparation for wisdom teeth extraction under general anesthesia. Her home medications included penicillin VK and regular intramuscular medroxyprogesterone acetate injections. Review of other systems was negative.

Initial studies (complete blood count, urine pregnancy test, urinalysis, and lumbar spine radiographs) were within normal limits (including hemoglobin and hematocrit of 11.3 g/dL and 32.5%, resp.). The patient was admitted for intravenous fluids and opioid analgesics; yet her pain remained poorly controlled. The patient was given azithromycin and ceftriaxone for uncomplicated cervicitis suggested by pelvic exam. She subsequently became febrile to 39.2°C and developed increasing drowsiness. Repeat laboratory studies revealed hemoglobin of 9.8 g/dL, hematocrit of 28%, and platelets of 91 × 10^9^/L and sodium of 127 mEq/L.

On hospital day 2, the patient developed dizziness, tachypnea, and mild hypoxia. Significant new laboratory findings included elevated aspartate amino transferase (248 U/L) and C-reactive protein of 215.2 mg/L (normal < 5 mg/L). Antibiotics were adjusted to cefepime and vancomycin, and the patient was transferred to the pediatric intensive care unit.

Head CT was normal and CT of the abdomen and pelvis was unremarkable except for small bilateral pleural effusions. At that time, meropenem, acyclovir, and doxycycline were added to the patient's antibiotic regimen. Due to progressive neurological and cardiovascular decline, the patient was endotracheally intubated and started on cardiovascular support. An echocardiogram revealed right-sided enlargement and elevated pressures (two-thirds of systemic circulation).

Thrombocytopenia as well as abnormal coagulation labs, including a D-dimer, suggested disseminated intravascular coagulopathy. Despite worsening anemia, hemoglobin electrophoresis obtained on the second day of hospitalization revealed a hemoglobin S of only 9.1% reflective of recent erythrocytapheresis procedure.

The patient's cardiovascular and neurological status further deteriorated with eventual loss of brainstem reflexes and EEG activity. On hospital day 7, she was declared brain dead. Results of serology testing returned postmortem showed parvovirus B19 PCR 300 copies/mL (normal < 100 copies/mL).

## 3. Autopsy Findings

**Figure 1:**
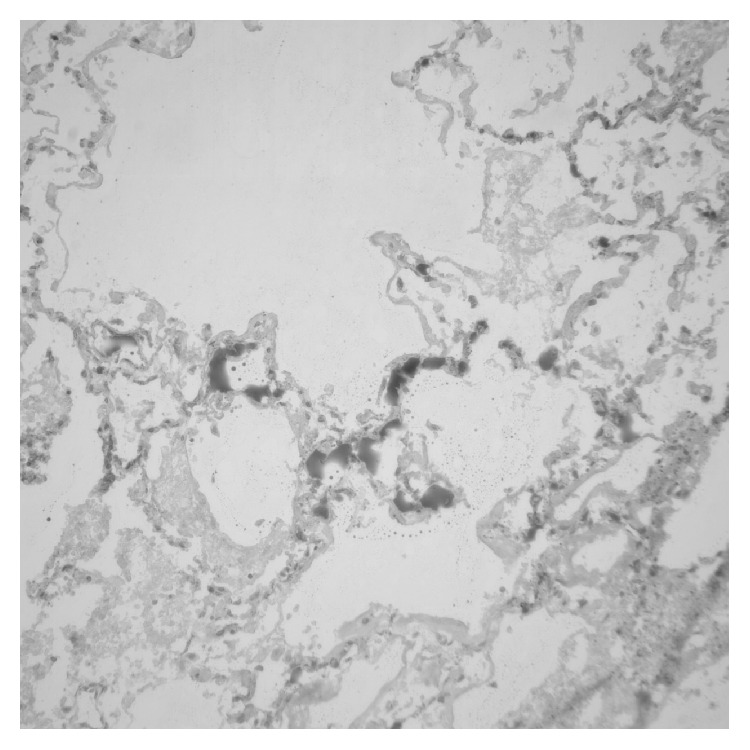
The lipid in the lung capillaries is staining positive with oil red O fat stain (20x original magnification).

**Figure 2:**
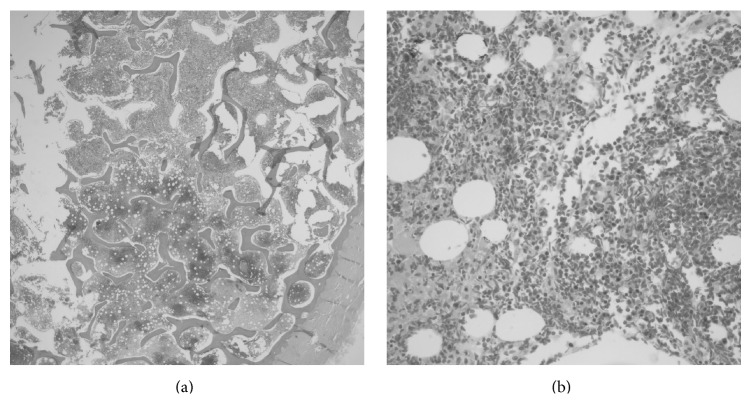
(a) The lower portion demonstrates the necrotic bone marrow compared to the upper viable marrow (2x original magnification, H&E stain). (b) The border of the viable and necrotic marrow shows sickling of red cells (40x original magnification, H&E stain).

**Figure 3:**
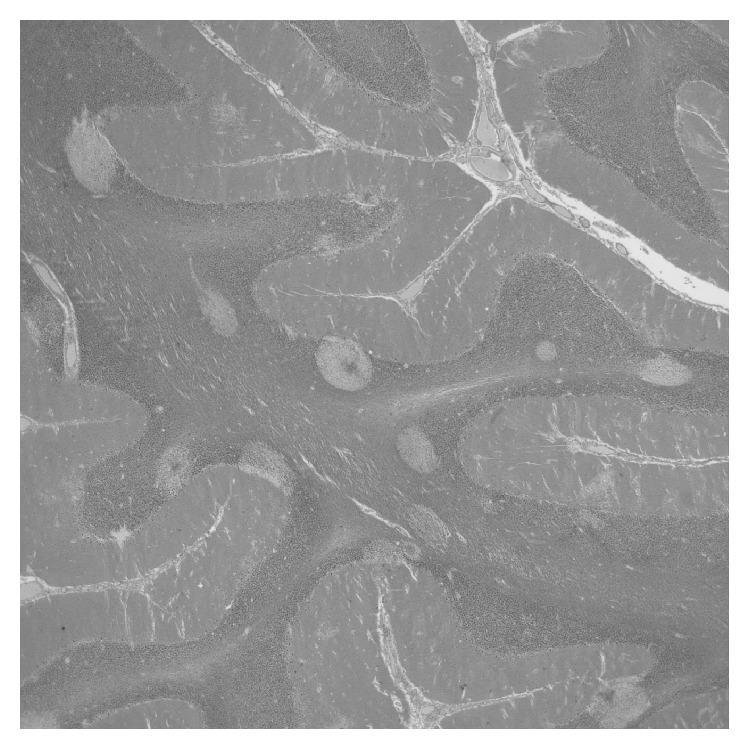
The pale ovals in the cerebellum were typical of the edematous foci throughout the central nervous system. There was often a small vessel in the center of the edema (2x original magnification, H&E stain).

Her findings were significant for enlarged and hemorrhagic lungs as well as an enlarged, infarcted spleen. The brain was edematous and dusky in appearance. All cultures were negative. On microscopic examination there were diffuse clear “bubbles” within the lungs, bone marrow, and all levels of the brain (spinal cord to cortex) (Figure 1). These “bubbles” stained on formalin fixed, frozen lung with fat stains (Figure 2). The viable areas of bone marrow showed clumps of sickled cells (Figure 3). The erythropoietic cells focally demonstrated parvovirus antigen on immunostains.

## 4. Discussion

Fulminant bone marrow necrosis leading to FES is not a well-understood subject but its association with an infectious etiology, specifically parvovirus B19, and SCD is becoming further evident. A previous study had shown that 91% of the patients with chronic hemolytic anemia with aplastic crisis and/or bone marrow necrosis had acute B19V infection [7, 8]. More importantly, none of the study candidates actually displayed traditional clinical* erythema infectiosum* symptoms [7, 8]. A lack of diagnostic tests makes it difficult to determine the actual incidence leading to FES in SCD patients.

At present, there are two current criteria classifications proposed for the diagnosis of FES. Gurd's classification requires at least one major and 4 minor criteria While Schonfeld's criteria require a cumulative score >5 for diagnosis [9] as shown below.

Gurd's and Schonfeld's criteria for classification of fat emboli syndrome are as follows.

Gurd's criteria [9] (1 major + 4 minor) are

 
*major*:
   axillary or subconjunctival petechiae,
   hypoxemia (PaO_2_ < 60 mmHg; FiO_2_ < 0.4),
   CNS depression,
   pulmonary edema;
 
*minor*:
   tachycardia lasting > 120 minutes,
   hyperthermia,
   retinal fat emboli,
   urinary fat globules,
   sputum fat globules,
   decreased platelet or hematocrit,
   increased erythrocyte sedimentation rate.

Schonfeld's criteria [9] (score > 5) are

 
*5 points*: petechiae,
 
*4 points*: chest X-ray changes,
 
*3 points*: hypoxemia (<9.3 kPa),
 
*1 point each*:
   fever > 38°C,
   tachycardia > 120 minutes,
   tachypnea > 30 minutes,
   confusion.

In regard to our patient, the bone marrow infarctions presumably accounted for the patient's pelvic pain. The pelvic bones likely also had marrow involvement similar to that in the lumbar vertebra based on her symptoms. The collateral blood supply of vertebral bodies is very limited, making this area fragile and susceptible to occlusions, which could further explain our patient's initial presenting symptom of lower back pain and findings of spinal artery occlusions on autopsy [10, 11]. The sudden onset of pulmonary and central nervous system symptoms was due to the widespread capillary embolization of lipid in the lungs and brain. This is a known complication of sickle disease with marrow infarction.

The patient had low levels of sickle cells in the blood and yet had compacted sickle cells in the marrow sinusoids that appeared to be the cause of the infarction, and these areas were marked by parvovirus infection.

Necrotic marrow emboli have been previously demonstrated in patients with acute chest syndrome and sickle cell anemia. One mechanism of thought is that large amounts of fat are able to cross the pulmonary vascular system and bypass smaller capillary systems [7, 12]. This would result in fat emboli entering the systemic circulation and affecting multiple different organ systems. It has been proposed that plasma lipids can alter their solubility when induced by a humoral factor seen with marrow necrosis, leading to intravascular coalescence of lipids [12]. On the other hand, the description of central nervous system lipid emboli diagnosed by MRI scans in trauma cases suggests that free lipid, not just marrow, can embolize in trauma. Unlike cellular marrow emboli that cannot pass through pulmonary capillaries, free lipid does traverse the lungs to reach the systemic circulation.

Previous studies also eluded that transfusion with packed red blood cells, without whole blood exchange, would not likely reverse fulminant fat embolism [13]. But, if diagnosed early in the clinical course, aggressive transfusion would prevent death and permanent end organ damage [13]. This was not the case with our patient. She was readily transfused with multiple units of packed red blood cells without reversal of any symptoms. This could be due to the advanced staging of her disease or may signify the limitations of simple transfusions for FES, which would need further clinical investigation.

There is currently no specific drug protocol for FES. High dose corticosteroids, with no standardized dosing, have been implemented for prevention of FES but show no conclusive benefits [9]. Other agents cited in literature include dextrans and hypertonic dextrose, aspirin, intravenous ethanol, and heparin [9]. These agents may exacerbate bleeding in the patients, further complicating the clinical course. There is a fine line between stabilizing and exacerbating symptoms in regard to volume status in these patients as well. Currently there are clinical trials evaluating a parvovirus immunization to help prevent FES but the findings are in early stages.

In regard to the route of transmission of parvovirus to our patient, it is unknown. Although case reports of B19V transmission by blood component transfusion are known with rates up to 1.4%, no studies have systematically determined a rate of transmission to recipients transfused with B19V DNA-positive components [14–17]. Tattooing has been suspected as a source of parvovirus infection and our patient had multiple tattoos two weeks prior to admission [18]. Since B19V is a common droplet-associated infection, it is also possible that the patient was infected naturally with the virus.

Our case emphasizes the importance of a high clinical suspicion of FES when a patient with sickle cell disease develops respiratory distress, neurologic syndromes, and anemia/thrombocytopenia in a setting of pain crisis. FES can be fatal complication in patients with HbSC disease. The need for more specific diagnostic tests and treatment protocols is urgent as this issue becomes more prevalent.
